# Seroprevalence of Dengue and Chikungunya antibodies among blood donors in Dar es Salaam and Zanzibar, Tanzania: a cross-sectional study

**DOI:** 10.1186/s12879-021-06549-y

**Published:** 2021-09-06

**Authors:** Haliya S. Shauri, Esther Ngadaya, Mbazi Senkoro, Joram J. Buza, Sayoki Mfinanga

**Affiliations:** 1 Nelson Mandela African Institute of Science and Technology Arusha, Arusha, Tanzania; 2 National Institute for Medical Research Muhimbili, Dar es Salaam, Tanzania

**Keywords:** Dengue, Chikungunya, Seroprevalence, Viruses, Tanzania

## Abstract

**Background:**

The potential shift of major causes of febrile illnesses from malaria to non-malarial febrile illnesses, including arboviral diseases such as chikungunya and dengue, is of concern. The last outbreaks of these infections were reported in 2018 and 2019 for chikungunya in Zanzibar and dengue in Dar es Salaam. We conducted a cross-sectional study that involved serological testing of stored blood samples from the blood banks in Temeke Referral Hospital in Dar es Salaam and the National Blood Bank Unit in Zanzibar. The samples were collected from Zanzibar and Dar es Salaam donors in May and June 2020, respectively. A total of 281 samples were included in the study, and their demographic information extracted from the registers. The samples were then transported to Muhimbili University of Health and Allied Sciences at the Microbiology Laboratory. They were subjected to an indirect ELISA to detect IgG and IgM against dengue and chikungunya viruses.

**Results:**

Seropositive IgM samples from Dar es Salaam were 3/101 (2.97%) for chikungunya and 1/101 (0.9%) for dengue, while samples from Zanzibar were all IgM negative for both viruses. Chikungunya IgG seropositivity was significantly higher (*p* ≤ 0.05) in Dar es Salaam 21/101 (21.2%) than Zanzibar 22/180 (12.2%). There was no difference in dengue IgG seropositivity between Dar es Salaam 44/101 (43.5%) and Zanzibar 68/180 (37.8%). Similarly, dual IgG seropositivity for both dengue and chikungunya viruses were not different between Dar es Salaam 13/101 (12.9%) and Zanzibar 11/180 (6.1%).

**Conclusion:**

Detection of IgM for dengue and chikungunya in Dar es Salaam indicates recent or ongoing transmission of the two viruses in the absence of a reported outbreak. These findings suggest the possibility of transmission of the two infections through blood transfusion. Detection of IgG antibodies for dengue and chikungunya viruses might be contributed by both; the ongoing infections and residual responses caused by preceding infections in the country. Results from blood banks may represent the tip of the iceberg. Further studies are needed to gain insight into the actual burden of the two diseases in Tanzania.

## Background information

Arboviral diseases, including dengue and chikungunya, are a certain public health concern in many tropical and sub-tropical countries [1, 2].The main vector of dengue and chikungunya is *Aedesaegypti.* However, *Aedesalbopictus* can harbor and transmit viruses [3, 4]. Also, the viruses may be directly transmitted through blood donation by asymptomatic donors [5]. Dengue and chikungunya viruses have similar transmission modes, same vector and pathological mechanisms, and clinical presentations [4]. The two febrile diseases are characterized by acute fever, high body temperature above 40^ °^C, muscle pain and headache, backache, and skin rashes [6]. Infection by any of the four dengue serotypes may be occurring with different clinical presentations and often with unpredictable clinical evaluation and outcome. Therefore, dengue case is classified as dengue fever with or without warning signs and severe dengue characterized by severe plasma leakage, severe hemorrhagic, and severe organ impairment [7]. In most cases, chikungunya is a self-limiting disease, though its complication, mainly joint pain, can persist for months or years post-infection, especially for older age [8, 9].

Arboviral disease transmission is often heterogeneous due to the vectors and host distribution and underlying social and ecological determinants [10]. The recent epidemics caused by these arboviruses have been associated with many factors, including urban expansions, population growth, and international travel and trade, which facilitate the spread of vectors and arboviruses into new niches amplification through the human-vector-human cycle [4]. Also, the areas with high temperatures and heavy rainfall followed by flooding are most favorable for mosquitoes' growth and survival [11]. Evidence of abundances of Aedes mosquitos has been documented in Zanzibar, where out of 200 samples, 124 (62%) were positive for immature stages of mosquitoes, of which 114 (94%) were positive for *Aedes*aegypti larvae and pupae [12]. In Tanzania mainland, a study conducted in the Morogoro region reported that immature *Aedes* mosquitoes were present in breeding sites during the rainy season (18.87%) and dry season (4.64%) [13]. Recent reports on dengue and chikungunya outbreaks show that diseases have spread in many parts globally, including Asia, the Pacific, Europe, and Africa [14–16]. Dengue outbreaks were reported in 2010, 2014, and more recently in 2018 in Tanzania [17]. The outbreak of 2019 was the worst documented dengue outbreak. Dar es Salaam was the epicenter, followed by Tanga with 6873 cases and 13 death reported [18]. The outbreak of chikungunya in Zanzibar was reported on 4th May 2018, with around 50 cases per day seen in MnaziMmoja referral Hospital [18]. Some studies have also documented chikungunya seroprevalence in different parts of Tanzania Mainland [20, 21].

Dengue cases associated with transfusions and transplantations have been reported [22, 23]. While these cases may not by themselves cause substantial public health alarm, but they may indicate possible future outbreak which may have huge public health and economic consequences. This study aimed to determine dengue and chikungunya’s seroprevalence in blood donors using the stored blood samples from Temeke referral hospital in Dar es Salaam and Zanzibar National Blood Bank in Zanzibar.

## Methodology

### Study site

This study was conducted in two areas of Tanzania; Dar es Salaam and Zanzibar. The archipelago of Zanzibar is a semi-autonomous region of Tanzania, situated in the Indian Ocean off the east cost of mainland Tanzania. The annual rainfall of Zanzibar is about 1600 mm in Unguja Island and 1900 mm in Pemba Island. Annual temperatures are high throughout the year, temperature range from 29 to 33 °C. Dar es Salaam is among the coastal regions of Tanzania, which lies 16 m above sea level with an average temperature of 26.1^ °^C/79.1 ^o^F and annual precipitation amount to 1150 mm.These conditions in the twolocations are more favorable for mosquitoes’ survival and growth. Since 2010, these areas have experienced several dengue and chikungunya cases [24].

### Study design

A cross-sectional study was conducted from May to October 2020. Blood samples and demographic information were retrieved from the Temeke Referral Hospital, Dar es Salaam and the Zanzibar National Blood Bank.

### IgG and IgM ELISA for detection of anti-dengue and anti-chikungunya antibodies

Serum was separated from whole blood by centrifugation and stored at  − 20 °C. All anti-dengue and anti-chikungunya were detected using indirect Enzyme-Linked Immunosorbent assays ELISA (Euro immune company from Germany).All assays were performed according to the manufacturers’ procedures, and all serum samples were diluted 1 into 100 with sample diluent provided with the kits. The optical density (OD) was measured at 450 nm, and the units of antibody concentration and cut-off values calculated as described by the manufacturers. Briefly, for the Anti-dengue IgM/IgG and IgM anti-chikungunya ELISAs the diagnostic cut-off value was calculated as the average OD of negative controls + 0.300. For the IgG chikungunya ELISA, the threshold for positivity was based on the OD cut-off value of the cut-of control + 10% [25].

### Statistical analysis

Data were retrieved from the computer then were compiled and analyzed using STATA v 15 software. Chi-square (*χ*^2^) was used to compare categorical data. The association between seroprevalence and the demographic variable was done using simple logistic regression and the odds ratio (OR) with 95% confidence intervals were estimated. Prevalence differences were considered to be statistically significant if *P* is ≤ 0.05 and if the 95% confidence does not include one.

### Informed consent

Informed consent was obtained from all subjects. All methods were carried out in accordance with declaration of Helsinki.

## Results

### Descriptive statistics

A total of 281 blood samples were tested, whereby 180 (64%) were from Zanzibar, and 101 (35.9%) were from Dar es Salaam. However, we could only retrieve demographic information of blood donors’ samples from Zanzibar. Out of 180 samples from Zanzibar, almost all blood samples, 171 (95%), were male donors. About 96 (53.3%) were in the age of < 30 years with a mean (SD) age of 37 (12.97) years. About 91 (50.6%) were unemployed people.

### Seroprevalence of Dengue and Chikungunya with their co-infections in Zanzibar and Dar-es-salaam

We detected dengue IgG seropositivity in both study sites; 43.5% (44 /101) in Dar es Salaam and 37.8% (68/180) in Zanzibar prevalence was not different. However, the chikungunya IgG prevalence in Dar esSalaam 21/101 (21.2%) was significantly higher (*P*-value = 0.047) than in Zanzibar 22/180 (12.2%). Neither dengue nor chikungunya seropositive IgM was observed in Zanzibar, while in contrast, both chikungunya IgM 3/101(2.97%) and dengue IgM 1/101(0.9%) were detected in Dar es Salaam. The prevalence of dual anti-chikungunya and anti-dengue IgG antibodies in the same sample was 13/101(12.9%) for Dar es Salaam and 11/180 (6.1%) in Zanzibar. However, the prevalence was not statistically different (P-value = 0.052). Table 1

**Table 1:** Seroprevalence of Dengue and Chikungunya with dual infection in Zanzibar (N = 180) and Dar-es-salaam (N = 101)

Test	Location	Positive n (%)	Confidence intervals	*P*-value
Dengue IgG	Zanzibar	68 (37.8)	31.0–45.0	0.342
	Dar-es-salaam	44 (43.5)	34.0–53.0	
Chikungunya IgG	Zanzibar	22 (12.2)	7.0–17	0.047*
	Dar-es-salaam	21 (21.2)	13–29	
Dengue IgM	Zanzibar	0 (0)	NA	NA
	Dar-es-salaam	1 (0.9)	− 1.0–3.0	
Chikungunya IgM	Zanzibar	0 (0)	NA	NA
	Dar-es-salaam	3 (2.97)	− 1.0–3.0	
Dual infection IgG	Zanzibar	11 (6.1)	3.0–10	0.052
	Dar-es-salaam	13 12.9)	6.0–20	

^a^Chi-square test comparing prevalence between Zanzibar and Dar-es-salaam

*P < 0.05, **P < 0.01, ***P < 0.001

### Risk factors for Dengue and Chikungunya

Different risk factors were evaluated, including age, sex, marital status, and occupation association with the two diseases. However, no risk factor was associated with any of the two diseases Table 2.

**Table 2:** Logistic regression of demographic factors associated with Dengue IgG, Chikungunya and dual-infection in Zanzibar (N = 180)

Variables	Dengue IgG			Chikungunya IgG			Dual infection of Dengue and Chikungunya		
	*n*	OR (95% CI)	*P-*value	*n*	OR (95% CI)	*P*-value	*n*	OR (95% CI)	*P*-value
Age									
19–30	38	Ref		11	Ref		5	Ref	
31–40	17	1.6(0.78–3.65)	0.183	6	1.78(0.61–5.25)	0.291	3	1.89(0.42–8.36)	0.400
41–61	13	0.89(0.41–1.94)	0.779	5	1.27(0.41–3.92)	0.678	3	1.68(0.38–7.40)	0.491
Sex									
Male	64	Ref		22	Ref		11	Ref	
Female	4	1.3(0.34–5.16)	0.673	0	NA	NA	0	NA	NA
Marital status									
Married	35	Ref		14	Ref		8	Ref	
Divorced	1	0.46(0.04–4.67)	0.517	0	NA		0	NA	
Single	32	0.74(0.41–1.37)	0.348	8	0.47(0.18–1.2)	0.116	3	0.32(0.08–1.25)	0.101
Occupation									
Not work	31	Ref		12	Ref		7	Ref	
Work	37	1.3(0.75–2.5)	0.300	10	0.83(0.34–2.04)	0.69	4	0.56(0.15–2.0)	0.376

## Discussion

The study was designed to compare the seroprevalence of anti-dengue and anti-chikungunya IgM and IgG and their co-circulation in Dar es Salaam and Zanzibar. We observed dengue and chikungunya IgM seropositivity of 0.9 and 2.97%, respectively, from Dar es Salaam samples, while no IgM for dengue or chikungunya was observed in samples from Zanzibar. Therefore, there is a need for screening for these infections and continued public education/awareness of avoiding exposure to Aedes mosquitoes. Due to the lack of routine diagnosis of these diseases, dengue and chikungunya fever could be misdiagnosed as malaria and wrongprescribsion given, leading to adverse health effects, especially for Dengue. Complications can lead to severe dengue infection characterized by severe plasma leakage, severe hemorrhage and organ impairment [7]. Again, IgM presence indicates that dengue and Chikungunya infections are ongoing in mainland Tanzania, and may be predictive of a future epidemic with serious social and economic consequences [7, 25, 26].

The lower chikungunya and dengue IgM seropositivity in this study is similar to the result obtained from a study conducted at Kilombero district in the South-Eastern part and Bondo district, the Northern part of Tanzania [29], [30]. In another study [21], the prevalence of chikungunya IgM was found to be (3.8%), which is relatively higher compared to our study. The differences could be attributed to many variables, including the type of samples used; our sample was collected from asymptomatic blood donors who could give different results if blood was taken directly from febrile patients or a random population sample. The random sample will provide an unbiased sample representing the entire population while a sample of blood donors has a high probability of being biased or unrepresentative of the population. Therefore, there is high probability for results to be different.

We observed dengue and chikungunya IgG seroprevalence in both Dar es Salaam and Zanzibar. This may be a consequence of ongoing infections as signified by IgM responses observed in this study and previous outbreaks [18], [31] since the IgG can be detected many years post-infection [32]. The anti-dengue and anti– chikungunya IgG antibodies detected in this study agree with many other studies in various parts of Tanzania [21, 27], [30], suggesting that the diseases are becoming endemic in the country.

The presence of chikungunya and dengue dual antibodies has been reported previously in Tanzania [20]. However, the reported estimates were lower thanthe prevalence reported in our study, which was 6.1% in Zanzibar and 12.9% in Dar es Salaam. The observed dual antibodies for chikungunya and dengue in the same individual in the study sites indicate that the two viruses are prevalent among blood donors. This may result in illness with overlapping signs and symptoms, which lead to difficulties in treatment and diagnosis.

No association was observed between seroprevalence and demographic characteristics. This might be due to a number of factors that represent the limitation of the study. To begin with, only a few demographic information was available from the blood bank register compared to what is normally collected during disease surveillance. Secondly, only samples from Zanzibar had the associated demographic data available for analysis, therefore, denying us the opportunity to compare the two locations. However, it is important to note that blood collection centers register only demographic information that is important for their purpose but not necessarily disease surveillance. Another limitation is that chikungunya and dengue viruses are not readily differentiated serologically due to cross-reactivity of their serocomplexes, so there is a need for molecular detection methods. However, the information obtained from this study will help to flag the potential danger of transmission of the two diseases through blood transfusion and also corroborate other studies suggesting that dengue and chikungunya may be endemic in Tanzania.

## Conclusion

This is the first study to document the seroprevalence of dengue and chikungunya in blood Bank in Dar es Salaam and Zanzibar. We recommend screening for both Dengue and chikungunya viruses infection for blood donors to avoid infection via transfusion, requiring viral detection in the form of RNA or antigen example, NS1. Also, we recommend a general population study in Dar es Salaam and Zanzibar to get a complete picture of the current disease burden in the country.

## Data Availability

The data used to support the findings of this study are available from the corresponding author (Shauri, Haliya) upon special request.

## Abbreviations

ELISA: Enzyme-Linked Immunosorbents Assay
SD: Standard Deviation
KNCHREC: Kibong’oto Infectious Diseases Hospital-Nelson Mandela African Institution of Science and Technology-Centre for Educational Development in Health, Arusha.
NS1: Non-Structure Protein 1
SUZA: The State University of Zanzibar
ZAHRI: Zanzibar Health Research Institute
